# Mapping Quantitative Trait Loci for High-Temperature Adult-Plant Resistance to Stripe Rust in Spring Wheat PI 197734 Using a Doubled Haploid Population and Genotyping by Multiplexed Sequencing

**DOI:** 10.3389/fpls.2020.596962

**Published:** 2020-11-12

**Authors:** Lu Liu, Congying Yuan, Meinan Wang, Deven R. See, Xianming Chen

**Affiliations:** ^1^Department of Plant Pathology, Washington State University, Pullman, WA, United States; ^2^Agriculture and Agri-Food Canada, Summerland Research and Development Centre, Summerland, BC, Canada; ^3^College of Life Sciences, Luoyang Normal University, Luoyang, China; ^4^Wheat Health, Genetics, and Quality Research Unit, Agricultural Research Service, U.S. Department of Agriculture, Pullman, WA, United States

**Keywords:** stripe rust, wheat, resistance, QTL mapping, doubled-haploid population, genotyping by multiplexed sequencing, yellow rust

## Abstract

Stripe rust caused by *Puccinia striiformis* f. sp. *tritici* (*Pst*) is a global concern for wheat production. Spring wheat cultivar PI 197734, of Sweden origin, has shown high-temperature adult-plant resistance (APR) to stripe rust for many years. To map resistance quantitative trait loci (QTL), 178 doubled haploid lines were developed from a cross of PI 197734 with susceptible AvS. The DH lines and parents were tested in fields in 2017 and 2018 under natural infection of *Pst* and genotyped with genotyping by multiplexed sequencing (GMS). Kompetitive allele specific PCR (KASP) and simple sequence repeat (SSR) markers from specific chromosomal regions were also used to genotype the population to validate and saturate resistance QTL regions. Two major QTL on chromosomes 1AL and 3BL and one minor QTL on 2AL were identified. The two major QTL, *QYrPI197734.wgp-1A* and *QYrPI197734.wgp-3B*, were detected in all tested environments explaining up to 20.7 and 46.8% phenotypic variation, respectively. An awnletted gene mapped to the expected distal end of chromosome 5AL indicated the accuracy of linkage mapping. The KASP markers converted from the GMS-SNPs in the 1A and 3B QTL regions were used to genotype 95 US spring wheat cultivars and breeding lines, and they individually showed different percentages of polymorphisms. The haplotypes of the three markers for the 1A QTL and four markers for the 3B QTL identified 37.9 and 21.1% of the wheat cultivar/breeding lines possibly carrying these two QTL, indicating their usefulness in marker-assisted selection (MAS) for incorporating the two major QTL into new wheat cultivars.

## Introduction

Global wheat (*Triticum aestivum* L.) production is challenged by stripe rust (also known as yellow rust), which is caused by fungus *Puccinia striiformis* Westend. f. sp. *tritici* Erikss. (henceforth *Pst*). Wheat stripe rust occurs more frequently in areas with cool and moist weather conditions during the wheat growing season (Chen, 2005) and has been reported in major wheat growing regions including the US, Western Europe, East and South Asia, the Arabian Peninsula, and Oceania (Wellings, 2011; Chen, 2020). Under favorable weather conditions, stripe rust can lead to complete yield losses on susceptible cultivars (Chen, 2005). Although fungicides can be applied to reduce yield losses caused by stripe rust, growing resistant wheat cultivars is the most efficient and environmentally sustainable way to control the disease (Line, 2002; Chen, 2013)

The stripe rust fungus is capable of producing a large number of races with virulence to different resistance genes (Wan and Chen, 2014; Liu et al., 2017). Resistance genes or quantitative trait loci (QTL) are classified into different types based on their effectiveness in different wheat growth stages and whether specific to different *Pst* races. All-stage resistance (ASR), also called seedling resistance, is effective only against specific *Pst* races at all growth stages. When deployed singly in cultivars, ASR can be easily defeated in a short period of time due to the emergence of new virulent races (Chen, 2005; Liu et al., 2019a). In contrast, adult-plant resistance (APR) is usually non-race specific, ineffective at seedling stage but expressed gradually as the plant ages. Most APR genes or QTL show enhanced resistance level at high temperatures and are therefore, termed high-temperature adult-plant resistance (HTAP) (Chen, 2013). APR or HTAP resistance is usually non-race specific and therefore, more durable than ASR. Individual genes or QTL for APR or HTAP resistance generally provide only partial protection, but commercially acceptable levels of resistance can be achieved by pyramiding multiple genes or QTL, which has been recommended for crop resistance breeding programs (Chen, 2013; Nelson et al., 2018). Although more than 80 formally designated *Yr* (for yellow rust) genes and hundreds of QTL have been identified for resistance to stripe rust of wheat (Maccaferri et al., 2015; Wang and Chen, 2017; Feng et al., 2018; Nsabiyera et al., 2018; Gessese et al., 2019; Pakeerathan et al., 2019; Li et al., 2020), many of them have already lost their effectiveness. Characterization of new resistance resources is important to achieve genetic diversity for resistance among new wheat cultivars.

Over the last 20 years, molecular markers utilized in wheat genotyping has shifted from simple sequence repeat (SSR) to single-nucleotide polymorphism (SNP) markers, which are the most abundant source of sequence variation within species (Rafalski, 2002). High-throughput SNP genotyping panels, such as the 90K Illumina iSelect wheat SNP chip and genotyping by sequencing (GBS) have been widely used in wheat genotyping. The 90K wheat SNP chip genotyping generates high-density SNPs with high quality (Wang et al., 2014) but has the limitation of relatively high cost compared with other technologies. GBS discovers SNPs across genomes at relatively low cost (Poland et al., 2012; Mascher et al., 2013). In addition, GBS is free of bias associated with a fixed array design such as 90K SNP chip, which is based on one set of populations but may not represent the SNPs in a new germplasm set. However, the set of GBS data may contain a large amount of missing data due to the low sequence coverage. Considering the limitations of these genotyping methods, a new PCR-based genotyping technology that uses highly multiplex SNP markers was applied in this study. This genotyping by multiplexed sequencing (GMS) protocol employs SNP markers selected from the 90K wheat SNP chip and additional known markers that are valuable in marker-assisted selection (MAS) for generating data points that cover the whole wheat genome (Ruff et al., 2020). Once resistance QTL are mapped with SNPs from GMS, SSR and kompetitive allele specific PCR (KASP) markers selected from specific chromosomal regions can be used to genotype the wheat population to validate and saturate the resistance QTL regions. This approach reduces the cost of genotyping and yields reliable results (Liu L. et al., 2020; Mu et al., 2020).

PI 197734 is a spring wheat cultivar, received from Sweden by the US Department of Agriculture, Agricultural Research Service, Small Grain Collections in 1951^1^. It had susceptible or intermediate reactions when tested with US predominant *Pst* races at seedling stage at low temperatures in the greenhouse but has shown a high level of resistance in fields for many years, indicating HTAP resistance to stripe rust (Wang M. et al., 2012). The objectives of the present study were to identify and map QTL for HTAP resistance to stripe rust in PI 197734 and test markers in the QTL regions for their usefulness in MAS.

## Materials and Methods

### Plant Materials

One hundred and seventy-eight doubled haploid (DH) lines were developed from the cross Avocet S (AvS) × PI 197734 following the procedure described by Zhang et al. (1996). This DH population was genotyped and tested in fields to map resistance QTL in PI 197734. In addition, a spring wheat panel of 95 US cultivars and breeding lines were used to test for the polymorphism of markers in the resistance QTL regions.

### Stripe Rust Phenotyping

Seedlings of AvS and PI 197734 were evaluated under controlled conditions in the greenhouse with predominant *Pst* races PSTv-4 (isolate 13–445), PSTv-14 (12–116), PSTv-37 (12–114), and PSTv-40 (09–78) (Wan and Chen, 2014). The virulence formulae of these *Pst* races on 18 *Yr* single-gene lines were provided in Supplementary Table S1. Five seeds of AvS and PI 197734 were planted in a 5 × 5 × 5 cm pot and were grown at 15–25°C. About 10 days after planting, seedlings at the two-leaf stage were uniformly dusted with urediniospores of each race mixed with talc at a 1:20 ratio (Chen and Line, 1992). Inoculated plants were placed into a dew chamber at 10°C and 100% humidity for 24 h without light and moved to a growth chamber with diurnal temperature cycle 4–20°C. Infection type (IT) was recorded about 18–20 days after inoculation using a 0–9 scale (Line and Qayoum, 1992). ITs 0–3 were considered as resistant; 4–6 intermediate; and 7–9 susceptible. Seedlings of the DH population were further tested with specific races that were avirulent to PI 197734 in the greenhouse to map QTL for the seedling resistance in PI 197734. Three seeds of each DH line, AvS, and PI197734 were planted in one well of 72-well trays. The growth conditions and inoculation method were the same as described above.

AvS, PI 197734, and the DH population were also tested for stripe rust response in the fields near Pullman in eastern Washington and Mount Vernon in western Washington in 2017 and 2018 under natural rust infection of *Pst*. In all these four location-year environments, DH lines were planted in a completely randomized block design with three replications. Each replication consisted of one row of AvS, PI 197734, and each DH line. About 50 seeds of each line were sown in a 50 cm row with 20 cm between rows. As a susceptible check, AvS was planted every 20 rows and surrounding the fields to ensure adequate and uniform *Pst* infection. Weed control and fertilization were applied following the local common practices. Stripe rust response was recorded as IT as described above and disease severity (DS), which is the percentage of infected area on flag leaves. The disease data were recorded at the heading-flowering stage when disease severity of AvS reached 90–100%.

### Statistical Analyses

Analyses of variance (ANOVA) were performed on the IT and DS data with the PROC MIXED procedure in SAS Statistical Software v9.1 (SAS Institute Inc., Cary, NC, United States) to partition variance components. Genotype (178 DH lines) was treated as a fixed effect, while environment (2 years in two locations) and genotype by environment interactions were treated as random effects. The broad-sense heritability (*H*^2^) was calculated as the ratio of the genetic variance ($\sigma_{G}^{2}$) to the total phenotypic variance ($\sigma_{p}^{2}$). The formula of phenotypic variance (*σ*_*G*_*p*2) is [$\sigma_{G}^{2}+$$\sigma_{G}\,E^{2}/m+\sigma_{e}^{2}/\left(m\,r\right)$], where *σ*_*G*_*E*^2^ is the genotype by environment interaction variance; $\sigma_{e}^{2}$ is the residual error variance; *m* is the number of environment; and *r* is the number of replication per environment (Piepho and Möhring, 2007). Pairwise Pearson’s correlation coefficients between the IT and DS data in each environment and among four environments were computed with the “rcorr” function in the R 3.5.1 Hmisc package^2^.

### Genotyping

Leaf tissue was harvested from one seedling plant of AvS, PI 197734, and each DH line at the two-leaf stage. Genomic DNA was extracted using the oKtopure^TM^ and sbeadex^TM^ plant nucleic acid extraction kit with an oKtopure^TM^ platform (LGC Genomics, Berlin, Germany). DNA was quantified using the Bio-Rad Fluorescent DNA Quantitation Kit (Bio-Rad Laboratories Inc., Hercules, CA, United States) on a BioTek Synergy 2 Microplate reader (BioTek, Winooski, VT, United States).

Genome-wide genotyping was performed with GMS and sequenced on an Ion Proton system (Life Technologies Inc., Carlsbad, CA, United States) based on the protocol developed by Ruff et al. (2020). Sorting, alignment, and SNP calling of sequence reads were performed as previously described (Ruff et al., 2020). After a resistance QTL was mapped, SNP markers from the 90K SNP array and SSR markers were selected from the specific chromosomal region, where the resistance QTL was mapped. These markers were first tested on the two parents and polymorphic markers were further used to genotype the DH population. SSR marker information was obtained from GrainGenes^3^ and MASWheat^4^, and the M13 tail (5′-CACGACGTTGTAAAACGAC) was added to the 5′ end of each forward primer of SSR markers. PCR were conducted as described by Hou et al. (2015), and products were electrophorized with an ABI3730 DNA fragment analyzer (Applied Biosystems, Grand Island, NY, United States). Alleles were scored using software GeneMarker v4.0 (SoftGenetics, LLC, State College, PA, United States). SNP markers were converted to KASP markers based on known primer information on CerealsDB^5^. FAM tail 5′-GAAGGTGACCAAGTTCATGCT and HEX tail 5′-GAAGGTCGGAGTCAACGGATT were added to allele-specific primers A and B, respectively. KASP assays were performed as described by Liu et al. (2019b). The end-point fluorescence data were visualized using a Roche Light-Cycler 480 real-time PCR system (Roche Applied Science, Indianapolis, IN, United States).

### Linkage Map Construction and QTL Analysis

Linkage construction was performed using JoinMap 4.1 (Van Ooijen, 2006). The regression mapping algorithm was used to assign linkage groups and Kosambi mapping function to calculate genetic distances in centiMorgans (cM). Markers that did not fit a 1:1 segregation ratio at *P* < 0.01 in a chi-squared test were excluded. Linkage groups were selected by a minimum LOD sore of 3.0 and were assigned to corresponding chromosomes based on the known chromosomal locations of SNP markers. Linkage maps were displayed with MapChart v2.3 (Voorrips, 2002).

Both IT and DS data from each of four field environments (Pullman, 2017, 2018; Mount Vernon, 2017, 2018) were used in QTL analyses. QTL analysis was conducted using QTL Cartographer 2.5 (Wang S. et al., 2012). The composite interval mapping (CIM) method with a walk speed of 1.0 cM and a window size of 10 cM were used to identify QTL. Significant LOD thresholds were calculated for each QTL using 1,000 permutations at *P* = 0.05 (Churchill and Doerge, 1994).

### Polymorphism of Markers Within QTL Regions

To determine the polymorphism of markers in the QTL regions, three KASP markers for the 1A QTL and four KASP markers for the 3B QTL were selected to test the spring wheat panel, including 95 US cultivars and breeding lines. KASP assays were performed as described above.

### Mapping of the Awnletted Gene

PI 197734 has short awns (awnletted) at the end of the spike, which is different from awned AvS. Based on this phenotypic difference, the awnletted gene in PI 197734 was mapped using the DH population to validate linkages constructed in this study. Awned or awnletted phenotypes were recorded for all DH lines planted in Pullman, WA in 2 years. This trait was input into QTL Cartographer 2.5 together with stripe rust responses to map the awnletted gene.

## Results

### Phenotypic Characterization of Stripe Rust Resistance

PI 197734 seedlings had an intermediate reaction (IT 6) to PSTv-37 and susceptible to PSTv-14 (IT 7), PSTv-4 (IT 8), and PSTv-40 (IT 8), while AvS were susceptible (IT 8) to all tested races. The DH population were tested with PSTv-37 to identify QTL for the intermediate reaction in PI 197734. However, only 8 of the 178 lines showed IT 6 and all other 170 lines had susceptible reactions (8 lines with IT 7; 162 lines with IT 8), which fit a 1:15 ratio for the segregation of intermediate and susceptible reactions (*P* = 0.33), indicating four loci in combination conferring the intermediate reaction. However, no significant QTL were detected in the QTL analysis, due to the small phenotypic differences among the DH lines. So, the further analyses were focused on the HTAP resistance in the field experiments.

**FIGURE 1:**
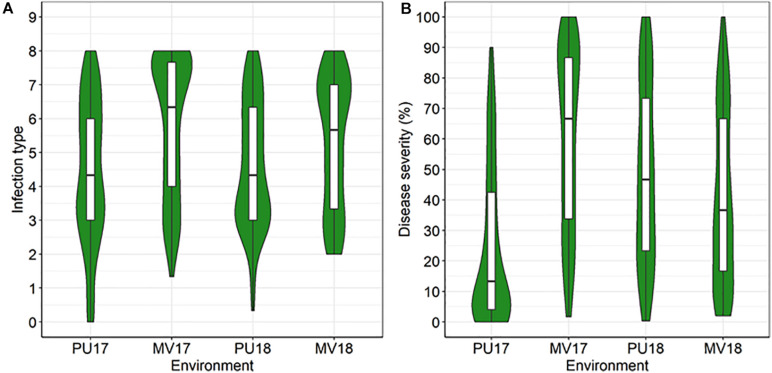
Violin plots for infection type **(A)** and disease severity **(B)** distributions of the AvS × PI 197734 doubled haploid (DH) population in response to stripe rust in four environments. PU17, MV17, PU18, and MV18 denote field experiments in Pullman (2017, 2018) and Mount Vernon (2017, 2018), respectively. The horizontal line displays the median. The top and bottom box edges display the first and third quartile values, respectively. The vertical lines represent the rest of the distribution. The green fill is a kernel density estimation to show the distribution shape of the data.

In all field tests, PI 197734 showed a high level of resistance at adult-plant stage. PI 197734 had IT 0–2 and DS 0–5% at Pullman, and IT 1–3 and DS 2–10% at Mount Vernon. The DH population showed continuous variations in IT and DS (Figure 1), indicating that the resistance in PI 197734 observed at adult-plant stage in fields was quantitatively inherited. Mean IT ranged from 4.4 to 5.8 and mean DS from 24.4 to 60.2% across four environments. Pearson’s correlation coefficients among all environments (Table 1) were significant (*P* < 0.0001), with *r* ranging from 0.78 to 0.85 for IT and from 0.68 to 0.85 for DS. As expected, IT and DS data in the same environments were highly correlated (*r* = 0.83–0.95).

**TABLE 1:** Correlation coefficients (*r*) of stripe rust infection type (IT) and disease severity (DS) across four environments.

**Environment^a^**	**PU17**	**MV17**	**PU18**	**MV18**
PU17	**0.83**^b^	0.68	0.74	0.73
MV17	0.79	**0.84**	0.83	0.77
PU18	0.78	0.79	**0.95**	0.85
MV18	0.80	0.80	0.85	**0.89**

*^*a*^The four environments were field tests in Pullman (2017) (PU17), Mount Vernon (2017) (MV17), Pullman (2018) (PU18), and Mount Vernon (2018) (MV18) under natural infection of the wheat stripe rust pathogen. ^*b*^r-values on the diagonal line (in bold) are for IT and DS comparison in the same environment; r-values under the diagonal line are for IT between different environments; r-values above the diagonal line are for DS between different environment. All correlations were significant at p < 0.0001.*

The ANOVA result showed that genetic, environment, and genetic by environment interaction had significant effects on stripe rust responses (*P* < 0.0001) (Supplementary Table S2). Broad-sense heritability (H^2^) was calculated as 0.94 using the IT data and 0.93 using the DS data, indicating the high heritability of stripe rust resistance in PI 197734.

### Construction of Linkage Maps

In total, 1,158 SNPs were generated by GMS, distributing over all 21 chromosomes. After filtering out markers with more than 30% missing data, 1,015 markers were retained, among which 303 were polymorphic. The genotype data of these 303 markers were input into Joinmap 4.1 for linkage construction. Markers that did not fit 1:1 segregating ratio (*P* < 0.01) were further excluded in Joinmap before linkage construction. With a LOD value of 3.0, 19 linkages were constructed, including 272 SNPs spanning 1705.7 cM. These linkage groups were assigned to corresponding chromosomes based on the chromosomal locations of SNP markers. No linkage groups were constructed for chromosomes 3D, 4D, and 6D; two linkages were constructed for 5B; and other 18 chromosomes were each represented by one linkage. The 5B-7B reciprocal translocation chromosome was detected according to the linkage maps. Additional 28 SSR and 30 KASP markers, which were selected from 1A and 3B chromosomes where the major resistance QTL were mapped, were used to determine their polymorphisms between the two parents. Among them, 8 SSR and 12 KASP markers were polymorphic and further used to genotype the DH population. Linkage construction was performed again with these markers, and all of markers, except two SSR markers, were included in the final linkages.

### QTL Analysis

Two major resistance QTL on chromosome 1A (*QYrPI197734.wgp-1A*) and 3B (*QYrPI197734.wgp-3B*) and one minor QTL on 2A (*QYrPI197734.wgp-2A*) were detected using the IT and DS data from four field trials (Table 2). *QYrPI197734.wgp-1A* were detected with both IT and DS data from all four environments and explained 6.73–20.70% phenotypic variation in different environments. *QYrPI197734.wgp-3B* provided the highest level of resistance and in 2017 at Pullman, it explained 46.80% phenotypic variation for the IT data. In other environments, the variation *QYrPI197734.wgp-3B* explained ranged from 14.45 to 33.25%. The minor resistance QTL, *QYrPI197734.wgp-2A*, was only detected with the IT data from Pullman and Mount Vernon in 2017 and the DS data from Mount Vernon in 2018, explaining 7.34–11.36% phenotypic variation. Additive effects of resistance QTL differed with QTL, data type, and different field trials.

**TABLE 2:** Quantitative trait loci (QTL) for stripe rust resistance detected in the AvS × PI197734 double haploid (DH) lines in field environments.

**QTL**	**Marker interval**	**Environment^a^**	**LOD^b^**	***R*^2^(%)^c^**	**AE^d^**
*QYrPI197734.wgp-1A*	*IWA2015*-*IWB6759*	PU17-IT	9.34	10.16	–0.67
		PU17-DS	5.58	6.73	–6.97
		MV17-IT	14.31	20.70	–0.93
		MV17-DS	4.73	9.07	–8.74
		PU18-IT	10.25	16.50	–0.76
		PU18-DS	9.55	19.75	–12.90
		MV18-IT	9.54	16.06	–0.78
		MV18-DS	10.08	17.93	–11.40
*QYrPI197734.wgp-3B*	*IWB10591*-*IWA6297*	PU17-IT	33.34	46.80	–1.45
		PU17-DS	22.18	33.25	–15.50
		MV17-IT	16.24	20.71	–0.92
		MV17-DS	10.48	17.58	–12.22
		PU18-IT	9.46	13.46	–0.68
		PU18-DS	9.07	16.14	–10.57
		MV18-IT	14.10	20.47	–0.88
		MV18-DS	8.87	14.45	–10.59
*QYrPI197734.wgp-2A*	*IWA5068*-*IWA5216*	PU17-IT	5.42	7.34	–0.57
		MV17-IT	7.03	10.62	–0.67
		MV17-DS	5.04	11.36	–9.78
		MV18-IT	4.73	9.45	–0.59

*^*a*^PU17, MV17, PU18, and MV18 denote field experiments in Pullman (2017, 2018) and Mount Vernon (2017, 2018), respectively. ^*b*^LOD, logarithm of odds score. ^*c*^R^2^-value is the percentage of phenotypic variance explained by individual QTL. ^*d*^AE, additive effect. A negative value indicates that the resistance allele is from PI 197734.*

The DH lines were grouped into eight subgroups containing different single QTL or QTL combinations based on the marker genotypes in the resistance QTL regions to determine the effects of QTL combinations. In general, more QTL in a combination provided higher resistance (Figure 4). As expected, lines with *QYrPI197734.wgp-3B* had lower disease ratings compared with lines with any of the other two single resistance QTL, indicating high resistance effect of *QYrPI197734.wgp-3B*. *QYrPI197734.wgp-2A* alone provided relatively low resistance, and stripe rust responses of lines with only *QYrPI197734.wgp-2A* were similar to lines with none of the three resistance QTL. However, when combined with one or more other QTL, *QYrPI197734.wgp-2A* contributed to an increased level of resistance. In general, lines with more QTL tended to have lower IT and DS values, or increased resistance, based on one-way ANOVA with the Tukey *Post hoc* test (*P* < 0.01). These results showed the primarily additive effects of the resistance QTL.

### Physical Maps of Resistance QTL and Alignment With Previously Reported Genes/QTL

**FIGURE 2:**
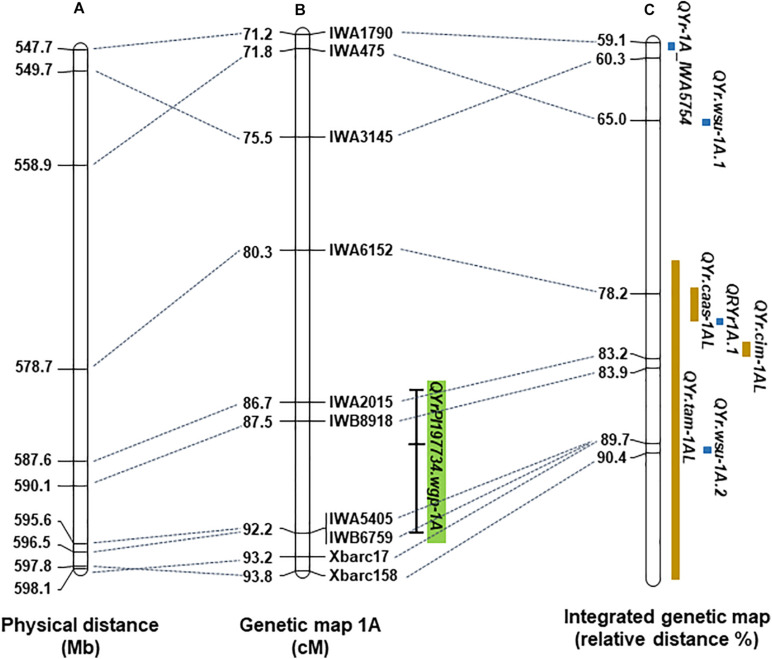
The position of *QYrPI197734.wgp-1A* on wheat chromosome 1AL. **(A)** The physical map of wheat chromosome 1AL according to the Chinese Spring IWGSC RefSeq v2.0 sequence. **(B)** The genetic linkage map of 1AL generated in this study and position of *QYrPI197734.wgp-1A* on the linkage map. The bar outlines QTL positions with a dash marking the position of the peak LOD score. **(C)** The integrated genetic map in Maccaferri et al. (2015) with previously mapped stripe rust resistance genes and QTL positioned on the map based on their linked markers. The chromosome length was standardized to a relative length. Resistance genes and QTL identified by linkage mapping are marked in blue color, while by association mapping are in brown color.

**FIGURE 3:**
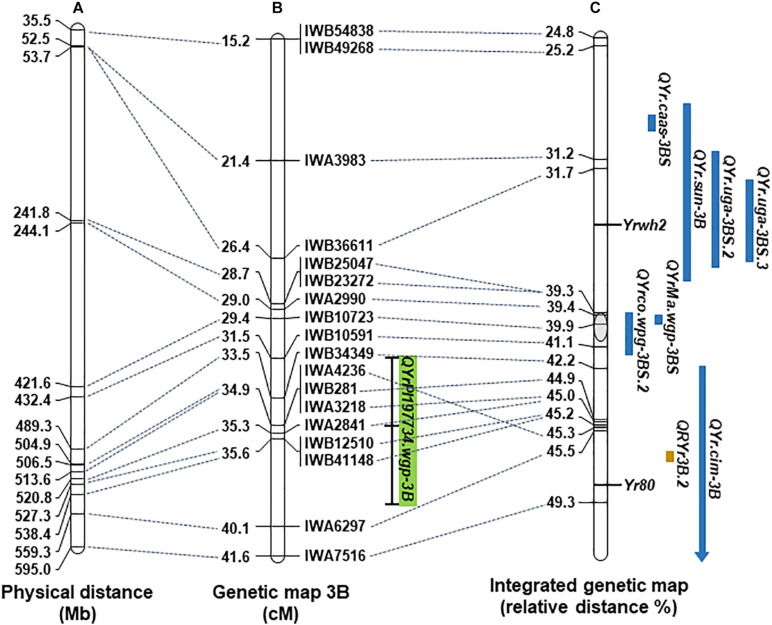
The position of *QYrPI197734.wgp-3B* on wheat chromosome 3B. **(A)** The physical map of wheat chromosome 3B according to the Chinese Spring IWGSC RefSeq v2.0 sequence. **(B)** The genetic linkage map of 3B generated in this study and position of *QYrPI197734.wgp-3B* on the linkage map. The bar outlines QTL positions with a dash marking the position of the peak LOD score. **(C)** The integrated genetic map in Maccaferri et al. (2015) with previously mapped stripe rust resistance genes and QTL positioned on the map based on their linked markers. The chromosome length was standardized to a relative length. Resistance genes and QTL identified by linkage mapping are marked in blue color, while by association mapping are in brown color.

The physical positions of markers in the two major resistance QTL, *QYrPI197734.wgp-1A* and *QYrPI197734.wgp-3B*, were obtained by aligning marker sequences with the Chinese Spring IWGSC RefSeq v2.0 sequence. Physical maps of selected portions of chromosomes 1A and 3B containing the resistance QTL, together with previously reported *Yr* genes or QTL, are shown in Figures 2, 3, respectively. Based on the alignments, *QYrPI197734.wgp-1A* was closely linked with previously reported stripe rust resistance QTL *QYr.caas-1AL*, *QRYr1A.1*, and *QYr.cim-1AL* and overlapped with *QYr.tam-1AL* and *QYr.wsu-1A.2* (Figure 2); and *QYrPI197734.wgp-3B* was closely linked with *QYrMa.wgp-3BS* and *QYrco.wpg-3BS.2*, and overlapped with *Yr80*, *QRYr3B.2*, and *QYr.cim-3B* (Figure 3).

### Polymorphism of Markers and Presence of QTL in US Spring Wheat

KASP markers (IWA2015, IWB8918, and IWA5405 for *QYrPI197734.wgp-1A*; IWB34349, IWB2841, IWB41148, and IWA6297 for *QYrPI197734.wgp-3B*) were used to genotype US spring wheat cultivars and breeding lines. All these markers showed considerable levels of polymorphisms among the US spring wheat cultivars and breeding lines (Supplementary Table S3). The polymorphic rates of the three KASP markers for *QYrPI197734.wgp-1A* were 22.2, 40.2, and 29.2%, respectively. The four KASP markers for *QYrPI197734.wgp-3B* were polymorphic at 36.8, 43.6, 60.0, and 43.2%, respectively.

When the three markers for *QYrPI197734.wgp-1A* have the same alleles as PI 197734 were considered to indicate the presence of the QTL, 36 (37.9%) cultivars or breeding lines possibly carry the QTL. Similarly, 20 (21.1%) cultivars or breeding lines possibly had the *QYrPI197734.wgp-3B* indicated by the same haplotype of the four KASP markers for the QTL (Supplementary Table S3).

### Mapping the Awnletted Gene

PI 197734 has short awns at the end of the spikes, which is different from AvS. As this trait has been previously mapped, it can be used to determine the mapping quality of the DH population, thus validating the QTL mapping for stripe rust resistance. The wide-type is awned, and three dominant loci *Tipped 1* (*B1*), *Tipped 2* (*B2*), and *Hooded* (*Hd*) have been identified to inhibit awn development in wheat, which are localized on chromosomes 5AL, 6BL, and 4AS, respectively (Yoshioka et al., 2017; DeWitt et al., 2020). Among them, *B1* is the most prevalent genotype inhibiting awn development and on its own, can produce short awns toward the top of the spike but absent elsewhere (Yoshioka et al., 2017; DeWitt et al., 2020; Huang et al., 2020). Recently, *B1* has been identified as the C2H2 zinc finger transcriptional repressor *TraesCS5A02G542800*, in the RefSeq 1.0 Chinese Spring reference genome from 698,528,636 to 698,529,001 bp on chromosome 5AL (DeWitt et al., 2020; Huang et al., 2020). In the present study, the awnletted gene was also mapped to the distal end of chromosome 5AL (Supplementary Figure 2), with the closest maker IWB47624 (679,657,926–679,658,026 bp in the Chinese Spring reference genome RefSeq 1.0), indicating of the accuracy of the linkage mapping.

## Discussion

The susceptible or intermediate reactions in the seedling stage tested at the low-temperature (4–20^*o*^C), but highly resistant reactions in field tests demonstrated that PI 197734 has a high-level of HTAP resistance to stripe rust. These results were in consistent with the previous report (Wang M. et al., 2012), indicating the stable resistance in PI 197734 over 15 years in the field. The whole-genome mapping using the field phenotypic data and GMS-SNP markers identified three QTL, *QYr197734.wgp-1A*, *QYr197734.wgp-2A*, and *QYr197734.wgp-3B*, on chromosomes 1AL, 2AL, and 3BL, respectively, for the HTAP resistance. KASP markers developed by converting SNP markers within the 1AL and 3BL QTL, which had stronger effects than the 2AL QTL, were validated using the AvS × PI 197734 DH population and a panel of spring wheat cultivars and breeding lines.

As PI 197734 has an intermediate reaction (IT 6) to race PSTv-37 at the seedling stage, the DH population was phenotyped in the greenhouse with this most predominant race of *Pst* in the U.S. (Wan and Chen, 2014; Wan et al., 2016). The segregation of the DH population for the intermediate reaction (IT 6) verses susceptible reaction (IT 7–8) indicating four loci involved. The number of the loci was not very different from the three QTL mapped using the field data. However, the QTL analysis using the greenhouse seedling IT data did not result in any significant QTL. This could be due that the difference between IT 6 and IT 8 is too small to find the involved genes. The intermediate reaction at the seedling stage could be from the relatively small effect of HTAP resistance in the early stage. From the breeding standpoint, the high level of HTAP resistance is more useful than the low-level resistance in the seedling stage. To incorporate the stripe rust resistance QTL from PI 197734 to elite breeding lines, progeny lines need to be tested in adult-plant stages.

Among the three QTL identified in the AvS × PI 197734 DH population, *QYr197734.wgp-3B* had the highest effect (Table 2). This QTL was mapped to the long arm of chromosome 3B (Figure 3), close to the centromere and within the confidence interval of *QYr.cim-3B* (Rosewarne et al., 2012) identified in ‘Pastor’, which is originated from the CIMMYT breeding program. However, the more detailed relationship between these two QTL cannot be established because *QYr.cim-3B* was mapped with DArT markers. *QRYr3B.2* (Jighly et al., 2015), identified in an association mapping, is also within the confidence interval of *QYr.cim-3B*. It was concluded to be the same QTL as *QYr.cim-3B* based on the QTL position and resistance source. *QRYr3B.2* is tagged by SNP marker IWA6510. However, the resistance allele information of IWA6510 for *QRYr3B.2* is not available. Thus, the QTL relationship of *QYr197734.wgp-3B* and *QYr.cim-3B* could not be established in the present study using haplotypes of multiple SNPs. *QYrPI197734.wgp-3B* are closely linked with *QYrMa.wgp-3BS* (Liu et al., 2018), *QYrco.wpg-3BS.2* (Case et al., 2014). As both *QYrMa.wgp-3BS* and *QYrco.wpg-3BS.2* were identified, respectively, in winter wheat cultivars Madsen and Coda developed in the US Pacific Northwest and likely to be the same QTL (Liu et al., 2018), *QYrPI197734.wgp-3B* may be different, but a further study is needed to clearly determine their relationship. *Yr80* for adult plant resistance was mapped in wheat landrace Aus27284 (Nsabiyera et al., 2018). As *QYr197734.wgp-3B* overlaps the *Yr80* locus (Figure 3), their relationship needs to be determined by testing PI 197734 and Aus27284 using the KASP markers of the two genes and also allelism testing.

*QYr197734.wgp-1A* is another QTL with a major effect. This QTL was mapped to the distal region of chromosome 1AL (Figure 2). *QYrPI197734.wgp-1A* is linked with *QYr.caas-1AL* (Ren et al., 2012), *QRYr1A.1* (Jighly et al., 2015), and *QYr.cim-1AL* (Rosewarne et al., 2012), and thus likely different from these QTL. *QYr.tam-1AL* (Basnet et al., 2014) and *QYr.wsu-1A.2* (Bulli et al., 2016) overlap with *QYr197734.wgp-1A*. *QYr.tam-1AL* is a minor QTL identified from the susceptible parent ‘TAM 112’, which makes it unlikely the same QTL as *QYr197734.wgp-1A*. *QYr.wsu-1A.2* was identified in an association mapping for a global winter wheat germplasm collection. It was tagged by SNP IWA3215 with the favorable allele G. To determine the relationship of *QYr197734.wgp-1A* and *QYr.wsu-1A.2*, KASP marker IWA3215 was tested on AvS and PI 197734. The nucleotide of AvS in IWA3215 was found to be A, and PI 197734 had G. Therefore, it is possible that *QYr197734.wgp-1A* and *QYr.wsu-1A.2* are the same QTL.

A minor-effect QTL, *QYr197734.wgp-2A*, was identified on the long arm of chromosome 2A, spanning about 30 cM on 2AL. The effect of this QTL was not stably detected across the field environments. Considering the small and unstable effect of *QYr197734.wgp-2A*, no KASP markers were developed for saturating this QTL region. Stripe rust resistance genes *Yr32* (Eriksen et al., 2004), *Yrxy2* (Zhou et al., 2011), one resistance QTL tagged by SNP marker IWA544 (Jighly et al., 2015), and *Qyr.saas-2A* (Yang et al., 2019) are located within the *QYr197734.wgp-2A* region. *Yr32* confers race-specific all-stage resistance to some *Pst* races. For example, it is resistant to races PSTv-4, PSTv-14, and PSTv-37, but ineffective against race PSTv-40 used in the present study (Supplementary Table S1). Similarly, the QTL tagged by IWA544 also confers all-stage resistance. Therefore, *QYr197734.wgp-2A* is different from these two genes or QTL. As *Yrxy2* is of HTAP type and *Qyr.saas-2A* is a minor QTL identified in field trials, their relationships with *QYr197734.wgp-2A* cannot be determined based on the current data.

In the present study, a new PCR-based genotyping approach, GMS, was used. At the time the DH population was genotyped, 1,158 SNPs were applied in the platform, while the current GMS platform multiplexes about 2,500 markers covering 2.7 cM per marker on average across the three genomes of hexaploid wheat (Ruff et al., 2020). GMS has the advantages of low cost, high accuracy, and ease at creating custom SNP panels to fit individual research needs. GMS has been previously applied in two association studies (Liu L. et al., 2020; Mu et al., 2020). Reliable linkages were constructed with relatively low number of markers in this study, as demonstrated by the accuracy in mapping an awnletted gene. Resistance QTL regions were further saturated by KASP and SSR markers. Thus, this process was proved to be an efficient way to map resistance genes in bi-parental populations.

**FIGURE 4:**
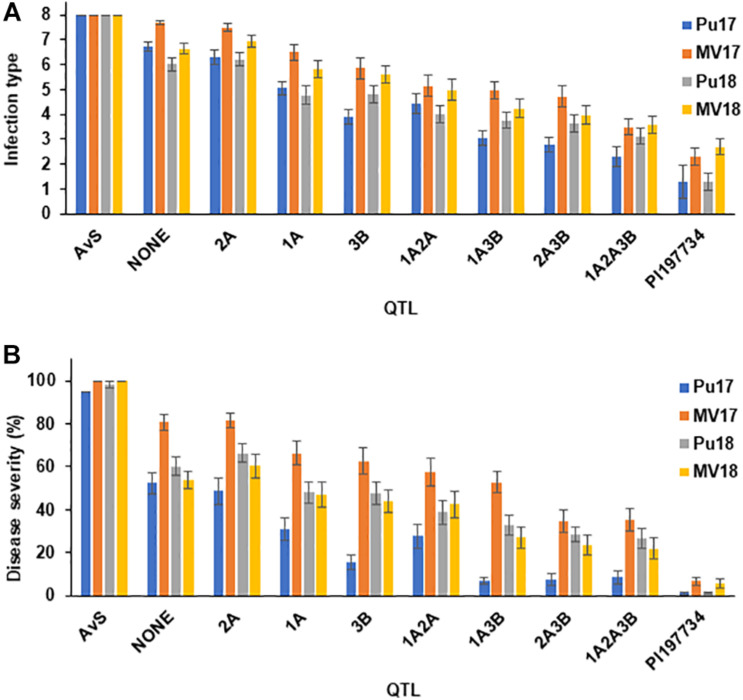
Infection type **(A)** and disease severity **(B)** ratings of doubled haploid lines containing different single and combinations of QTL for resistance to stripe rust in different field environments. PU17, MV17, PU18, and MV18 denote field experiments in Pullman (2017, 2018) and Mount Vernon (2017, 2018), respectively.

The mean IT and DS values of the DH lines grouped by markers for the three mapped QTL into different categories showed not only different effect levels of the three individual QTL, but also the levels of different combinations (Figure 4). In general, the QTL showed additive effects. DH lines with more QTL showed higher resistance, demonstrating that pyramiding multiple HTAP QTL each with partial resistance can improve the level of resistance, even to the high level in PI 197734, without producing any uredinia. The results agree with previous studies that combining different genes for HTAP resistance can achieve high level, durable resistance to stripe rust (Liu et al., 2018, 2019b; Liu L. et al., 2020; Mu et al., 2020).

A single marker linked to a resistance gene is often inadequate for MAS. Haplotypes of two or more markers can greatly improve the accuracy for MAS. The haplotype approach has been used in previous studies to indicate the presence and absence of resistance genes or QTL (Feng et al., 2015, 2018; Xiang et al., 2016; Liu et al., 2018, 2019b; Liu Y. et al., 2020). In the present study, we genotyped 95 spring wheat cultivars and breeding lines developed and grown in the U.S. Pacific Northwest with three KASP markers for *YrQPI197734-1A* and four KASP markers for *YrQPI197734-3B* converted from the GMS-SNP markers within the QTL regions (Supplementary Table S3). The individual markers showed considerable polymorphisms among the cultivars/breeding lines and identified 37.9% and 21.1% of the cultivars/breeding lines possibly carrying the 1A and 3B QTL, respectively, by their identical haplotypes with the alleles in PI 197734. As these cultivars/breeding lines have various levels of resistance to stripe rust (Supplementary Table S3), the presence of these QTL can be supported by their resistance phenotypes. The relatively high frequencies of these resistance QTL in U.S. spring wheat cultivars are not surprising as the Sweden wheat cultivar was introduced to the U.S. almost 70 years ago^6^ and it might have been used in U.S. breeding programs. The contemporary U.S. wheat cultivars/breeding lines may be easier for use to incorporate either the 1A QTL or the 3B QTL into new cultivars. However, none of the tested cultivars/breeding lines carrying both QTL based on the identical haplotypes of the three or four markers. Therefore, PI 197734 may be used for crossing with elite U.S. lines without the QTL if both QTL are to be incorporated. The haplotypes of the marker combinations can be used in MAS for developing new cultivars with these QTL or in combination with other genes/QTL for effective resistance to stripe rust.

## Conclusion

Wheat cultivar PI 197734 has a stable, high-level of HTAP resistance to stripe rust. A DH population was developed for studying the genetic basis of the stripe rust resistance in PI 197734. Using the combination of multi-environment phenotyping and GMS successfully identified two major-effect QTL on the long arms of chromosomes 1A and 3B and a minor-effect QTL on the long arm of chromosome 2A. The expected chromosomal location of mapping an awnletted gene in PI 197734 validated the accuracy of the linkage mapping. Categorizing the DH lines by the presence of markers for the three QTL and mean stripe rust responses showed additive effects, supporting the concept of pyramiding different genes for achieving high-level, durable resistance. The presence of the resistance haplotypes of the two major-effect QTL in relatively high numbers of US spring wheat cultivars/breeding lines indicated that the PI 197734 resistance to stripe rust has been incorporated into US wheat. The results indicate that the KASP markers converted from the GMS-SNPs in the resistance QTL regions can be used in MAS for incorporating the QTL into new wheat cultivars.

## Author’s Note

Mention of trade names or commercial products in this publication is solely for the purpose of providing specific information and does not imply recommendation or endorsement by the U.S. Department of Agriculture. USDA is an equal opportunity provider and employer.

## Data Availability Statement

The original contributions presented in the study are included in the article/Supplementary Material, further inquiries can be directed to the corresponding author.

## Author Contributions

LL conducted experiments, analyzed, interpreted data, and drafted and revised the manuscript. CY and MW participated in the experiments. DS provided resources and technique guidance. XC developed and guided the project, interpreted data, and wrote the manuscript. All authors reviewed the manuscript.

## Conflict of Interest

The authors declare that the research was conducted in the absence of any commercial or financial relationships that could be construed as a potential conflict of interest.

## Footnotes

1 https://npgsweb.ars-grin.gov/gringlobal/accessiondetail.aspx?id=1168330

2 https://CRAN.R-project.org/package=Hmisc

3 http://wheat.pw.usda.gov

4 http://maswheat.ucdavis.edu

5 http://www.cerealsdb.uk.net/cerealgenomics/CerealsDB/KASP_primers_for_iSelect.php

6 https://npgsweb.ars-grin.gov/gringlobal/accessiondetail.aspx?id=1168330
