# Supplementary figures and images for: Mapping Quantitative Trait Loci for High-Temperature Adult-Plant Resistance to Stripe Rust in Spring Wheat PI 197734 Using a Doubled Haploid Population and Genotyping by Multiplexed Sequencing
Source: Front Plant Sci. 2020 Nov 12;11:596962. doi: 10.3389/fpls.2020.596962 (PMC7688900; doi:10.3389/fpls.2020.596962)

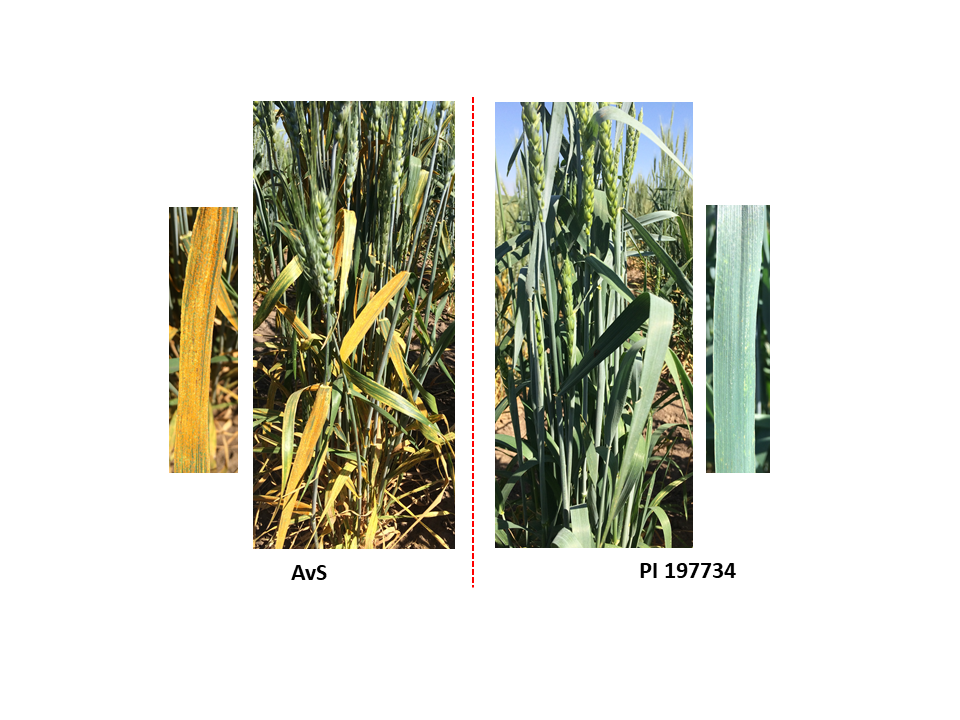

Supplement: Supplementary file 1 [file Image_1.TIF]

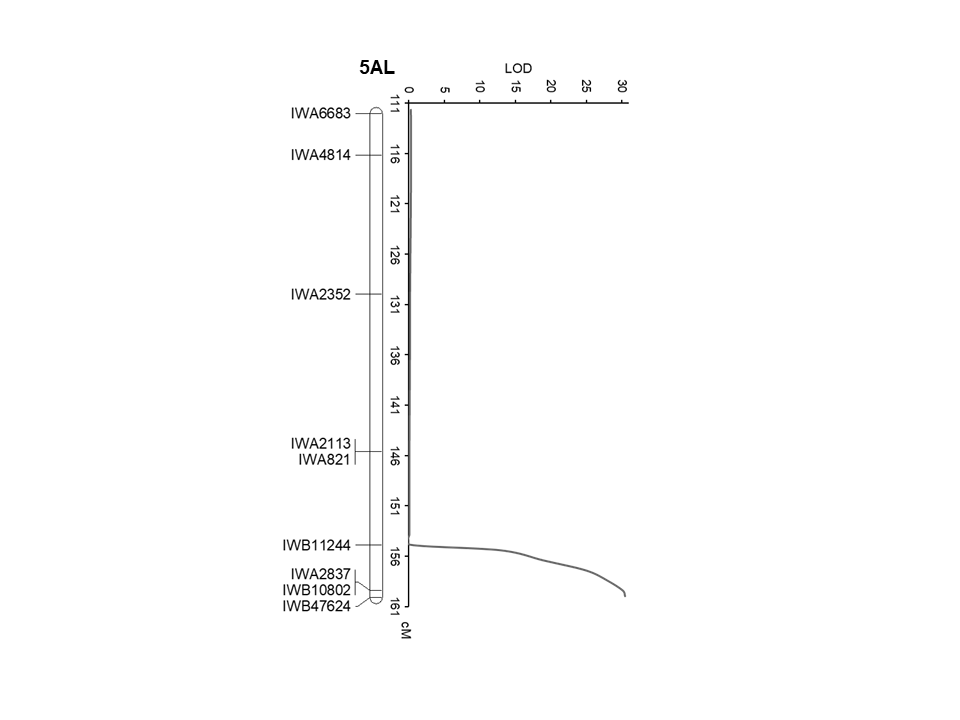

Supplement: Supplementary file 2 [file Image_2.TIF]
