# Supplementary material for: Mapping Quantitative Trait Loci for High-Temperature Adult-Plant Resistance to Stripe Rust in Spring Wheat PI 197734 Using a Doubled Haploid Population and Genotyping by Multiplexed Sequencing
Source: Front Plant Sci. 2020 Nov 12;11:596962. doi: 10.3389/fpls.2020.596962 (PMC7688900; doi:10.3389/fpls.2020.596962)
Supplement: Supplementary file 3 [file Table_1.DOCX]

**Supplementary Table S1** Virulence formulae and octal codes of four *Puccinia striiformis* f. sp. *tritici* races used in the seedling test

| Race | Octal code | **Virulence**/avirulence formula^a^ |
| --- | --- | --- |
| PSTv-4 | 511211 | **1,6,9,17,27,SP,76**/5,7,8,10,15,24,32,43,44,Tr1,Exp2 |
| PSTv-14 | 571267 | **1,6,7,8,9,17,27,43,44,Tr1, Exp2,76**/5,10,15,24,32,SP |
| PSTv-37 | 171266 | **6,7,8,9,17,27,43,44,Tr1,Exp2**/1,5,10,15,24,32,SP,76 |
| PSTv-40 | 174766 | **6,7,8,9,10,24,27,32,43,44,Tr1,Exp2**/1,5,15,17,SP,76 |

^a^ The octal codes and virulence formula were based on the 18 *Yr* single-gene differentials: *Yr1*, *Yr5*, *Yr6*, *Yr7*, *Yr8*, *Yr9*, *Yr10*, *Yr15*, *Yr17*, *Yr24*, *Yr27*, *Yr32*, *Yr43*, *Yr44*, *YrSP*, *YrTr1*, *YrExp2*, and *Yr76* (Wan and Chen 2014).
