# Supplementary material for: Mapping Quantitative Trait Loci for High-Temperature Adult-Plant Resistance to Stripe Rust in Spring Wheat PI 197734 Using a Doubled Haploid Population and Genotyping by Multiplexed Sequencing
Source: Front Plant Sci. 2020 Nov 12;11:596962. doi: 10.3389/fpls.2020.596962 (PMC7688900; doi:10.3389/fpls.2020.596962)
Supplement: Supplementary file 4 [file Table_2.DOCX]

**Supplementary Table S2** Analysis of variance of infection type (IT) and disease severity (DS) data for the AvS$\times$ PI 197734 doubled haploid population

| **Source of variation** | **IT** | | | |  | **DS** | | | |
| --- | --- | --- | --- | --- | --- | --- | --- | --- | --- |
|  | ***df*** | **MS** | **F** | ***P*** |  | ***df*** | **MS** | **F** | ***P*** |
| RILs | 177 | 39.4 | 16.7 | <0.0001 |  | 177 | 7639 | 14.2 | <0.0001 |
| Environments | 3 | 225.3 | 95.5 | <0.0001 |  | 3 | 120770 | 224.9 | <0.0001 |
| RILs*Environment | 530 | 2.4 | 3.4 | <0.0001 |  | 530 | 537 | 3.8 | <0.0001 |
| Residuals | 1420 | 0.7 |  |  |  | 1420 | 140 |  |  |
